# The Digital Therapeutic Alliance With Mental Health Chatbots: Diary Study and Thematic Analysis

**DOI:** 10.2196/76642

**Published:** 2025-10-10

**Authors:** Zian Xu, Yi-Chieh Lee, Karolina Stasiak, Jim Warren, Danielle Lottridge

**Affiliations:** 1 University of Auckland Auckland New Zealand; 2 National University of Singapore Singapore Singapore

**Keywords:** mental health chatbot, digital therapeutic alliance, longitudinal study, diary study, user experience, human-chatbot relationship, well-being, conversational agent

## Abstract

**Background:**

Mental health chatbots are increasingly used to address the global mental health treatment gap by offering scalable, accessible, and anonymous support. While prior research suggests that users may develop relationships with these chatbots, the mechanisms and individual differences underlying such relational experiences remain underexplored. As the concept of the digital therapeutic alliance (DTA) gains traction, a deeper understanding of subjective relationship-building processes is essential to inform the design of more effective digital mental health interventions.

**Objective:**

This study aimed to investigate how people subjectively perceive and develop relationships with mental health chatbots over time. We sought to identify key experiential dimensions and interactional dynamics that facilitate or hinder the formation of such bonds, contributing to the evolving conceptualization of the DTA.

**Methods:**

We conducted a 4-week short-term longitudinal diary study with 26 adult participants who interacted with two widely available mental health chatbots (Woebot and Wysa). Data were collected through weekly surveys, conversation screenshots, and semistructured interviews. A reflexive thematic analysis was used to identify recurring themes and interpret the emotional, communicative, and contextual factors shaping participants’ relational experiences with the chatbots.

**Results:**

A total of 18 participants reported forming a bond or light bond with at least one chatbot. Interview narratives revealed three relational categories: Bond (clear emotional connection), Light Bond (tentative or partial connection), and No Bond (absence of connection). Both participants with lower and higher psychological well-being (based on the World Health Organization—Five Well-Being Index scores) reported forming such relationships, suggesting that bonding capacity is not strictly dependent on mental health status. Thematic analysis identified six key themes that explain why people did or did not form bonds: the desire to lead or be led in conversation, alignment between preferred style of self-expression and accepted inputs, expectations for caring and nurturing from the chatbot, perceived effectiveness of the chatbot’s advice and proposed activities, appreciation for colloquial communication, and valuing a private and nonjudgmental conversation.

**Conclusions:**

Our findings provide empirical insight into how people interpret and engage in relational processes with mental health chatbots, advancing the theoretical foundation of the DTA. Rather than favoring one design style, our analysis highlights the importance of alignment between preferences and the chatbot’s interaction style and conversational role. Participants’ initial expectations around empathy and trust also shaped how relationships developed. Drawing on these insights, we suggest that chatbots may better support early therapeutic relationships by blending emotional support with relevant guidance, allowing flexible input methods, and maintaining continuity through context-aware responses. These features may enhance their therapeutic value and foster stronger relationships.

## Introduction

### Background

Mental health chatbots are an increasingly popular digital intervention, leveraging artificial intelligence (AI) to engage people in conversations that support their mental well-being [1-3]. In traditional psychological treatment, the relationship between a client and a therapist, known as the therapeutic alliance (TA), is a key factor in its success [4-6]. Researchers have begun investigating whether the concept of TA can also be applied to digital mental health tools, such as meditation apps [7] and mental health chatbots [1-3], leading to the emergence of the concept of digital therapeutic alliance (DTA) [8]. The emerging concept of DTA provides a new lens to examine the aspects that influence relationship-building, and ultimately, the success of digital mental health interventions. Researchers have increasingly focused on how DTA might affect therapeutic outcomes, drawing parallels to traditional TA [9-12]. Yet, how DTA is conceptualized, especially in relation to people’s sense of connection with mental health chatbots, if any, remains inconclusive [13-15], in particular due to the absence of longitudinal studies examining how relationships with mental health chatbots evolve over time [16].

To address this gap, this study explores perceptions of relationship development between people and chatbots. We conducted a diary study combined with semistructured interviews to capture a comprehensive view of interactions with mental health chatbots over a 4-week period. We recruited 26 participants who interacted with two prominent mental health chatbots, Woebot [1,3] and Wysa [17]. Through thematic analysis of an extensive dataset of transcripts and screenshots, we identified six main themes related to participants’ relationships with these chatbots.

Our findings emphasize six key aspects of human-chatbot interactions that impact humans’ relationship development with chatbots. Participants expressed a desire for flexibility in conversation, either leading the dialog or being guided by the chatbots. The ability to self-express, whether through free-text input or selecting from provided prompts, impacted humans’ relationships with chatbots. Additionally, people had specific expectations for the chatbot to provide caring and nurturing responses, along with effective advice and activities that were perceived as genuinely helpful. The appreciation for colloquial communication further strengthened the connection, while the importance of maintaining a private, nonjudgmental space was consistently highlighted as essential for building trust and fostering meaningful engagement.

As far as we know, this is the first study to use a diary method to qualitatively explore relationship development and identify the themes influencing DTA in the context of mental health chatbots [16,18]. This research advances the field of digital mental health by offering insights into how humans build relationships with mental health chatbots, by expanding the DTA framework, and by highlighting how chatbot and interaction design characteristics can foster stronger connections with people.

### Related Work

#### Therapeutic Alliance

TA refers to the relationship between clients and therapists [5,6,19]: its acceptance, empathic understanding, and congruence [20]. Studies on the working alliance [21-23] emphasize the client and therapist bond and agreement on therapeutic tasks and goals [24]. The Agnew Relationship Measure (ARM) emphasizes five dimensions of alliance: partnership, bond, confidence, openness, and client initiative [11,25]. The TA, referring to the collaborative and affective bond between client and therapist, is a well-established predictor of psychotherapy outcomes. A large-scale meta-analysis of nearly 300 studies found a moderate but robust correlation between alliance quality and treatment success (*r*=0.278) [4], with this association replicated across diverse contexts and sustained across decades of research [4,26-29]. Effective communication is recognized as a critical skill for clinicians; it improves the quality of care and strengthens the TA [30]. Research on relational control in physician-patient communications reveals that balancing control—where patients can take the lead, physicians manage control when necessary, and both share control to facilitate planning—enhances communication and relationship building in clinical settings [31-34]. This highlights the importance of two-way communication and the extent to which clients’ sense of control—or a lack thereof—during interactions impacts the development of their relationship with the therapists [35,36].

#### Digital Therapeutic Alliance

There is growing evidence for the positive effects of digital tools in helping clients with mental health challenges, and there is increasing interest in stand-alone digital solutions to augment or substitute traditional therapeutic methods [1,9,17]. However, uncertainties remain. Digital solutions might not be appropriate for individuals with severe mental illnesses [11], with conditions like depression impacting use [10]. Given that the TA has proven highly effective in face-to-face psychotherapy, theory on “DTA” is emerging within the scope of digital mental health intervention research. Researchers are actively studying how the concept of TA applies to digital mental health solutions and examining effects on therapeutic outcomes [9-12].

In 2019, the first symposium on the DTA took place in Melbourne [37]. The event addressed various forms of digital mental health care, including web or mobile apps, teletherapy, and AI-based therapy agents [12]. Some researchers emphasized the role of human-computer interaction (HCI) in establishing standards for DTA, identifying key HCI knowledge that could shape the formation of DTA: namely, persuasive system design, positive computing, and affective computing [38]. Canonical measures for TA were revised to measure the DTA: mobile ARM (mARM) through the lens of HCI [38]. HCI further contributed to understanding DTA by presenting methods for developing and integrating therapeutic content into digital interventions and evaluating their effectiveness [8,39,40]. Though DTA shows promise for improving digital mental health interventions, its concept remains underdeveloped, with limited research exploring its definition and predictive value [37,41]. Addressing this gap, Malouin-Lachance et al [42] conducted an integrative review that synthesized 28 studies to propose a conceptual framework for the DTA, highlighting core components such as goal alignment, task agreement, therapeutic bond, and user engagement. Their review underscores the potential of AI-driven tools—particularly chatbots—to replicate certain relational mechanisms found in traditional therapy, while also noting ongoing challenges related to personalization, ethical use, and long-term engagement. This framework provides a theoretical basis for understanding user-chatbot relationships and informs this study’s exploration of how people perceive and articulate emotional bonds in real-world interactions with mental health chatbots.

Past research on DTA has primarily relied on adapted or newly developed instruments to evaluate alliance quality in technology-mediated interventions. One widely used tool is the Working Alliance Inventory (WAI)-Short (WAI-S), which assesses the traditional therapeutic bond between clients and therapists across task, goal, and emotional connection dimensions [23]. To evaluate alliance in technology-mediated therapy, the Digital WAI (D-WAI) extends the original WAI framework to digital contexts. It includes two items each for task agreement, goal alignment and emotional bond, rated on a 7-point scale (1=strongly agree to 7=strongly disagree), with lower scores indicating stronger alliance [7,43]. In contrast, mARM was developed for unguided app-based interventions. It comprises 25 items across five dimensions: bond, partnership, confidence, openness, and client initiative, each rated on a 7-point Likert scale. A score of 4 represents a neutral stance, with higher scores reflecting a stronger perceived alliance [25].

While these tools offer structured and standardized assessments, they may not fully capture the early-stage, dynamic, or moment-based relational states typical of fully automated, memory-limited chatbot interactions. These limitations suggest the need for complementary approaches that more directly capture lived experiences and nuanced perceptions of alliance in chatbot-mediated contexts. In this study, rather than applying standard alliance instruments, we adopted an inductive, qualitative approach to explore how users describe and interpret relational dynamics over time. This allowed us to examine how therapeutic relationships emerge, fluctuate, and at times remain ambiguous in short-term interactions with unguided mental health chatbots.

#### Mental Health Chatbots and Human-Chatbot Relationship

Chatbots are now widely used for mental health support. A scoping review identified 41 chatbots across 53 studies, including Woebot and Wysa, which are used for various purposes such as delivering therapy (eg, cognitive behavioral therapy for depression and anxiety), training social skills, and screening for conditions like depression and dementia [2]. Most of these chatbots were rule-based, using written language for input and combining written, spoken, and visual outputs [2]. There is growing evidence for the positive effectiveness of mental health chatbots in psychological treatment [1,3,17,44,45].

To better understand the aspects influencing relationships with mental health chatbots, we review literature on human-chatbot relationships, particularly in the context of nonclinical social chatbots. Key aspects such as trust [46], self-disclosure [47,48], diverse interaction [49], human-like conversations [50,51], and interactivity [52,53] enhance subjective experiences of the relationship. Studies suggest that longer and more intense interactions with chatbots lead to stronger feelings of social connectedness [54]. These aspects may also be relevant in clinical domains, yet health care chatbots remain understudied [54].

Although few studies on mental health chatbots directly consider the impact of relationships, they have identified elements that contribute to positive user experiences, such as usefulness, ease of use, responsiveness, understandability, and acceptability [55], as well as trust and enjoyment [14]. These elements help build intimacy with chatbots, boosting satisfaction and reuse intentions [15,55]. Multiple studies and a recent systematic review [16] have identified several design features that foster connectedness in AI-driven mental health chatbots. These include diverse content (eg, videos and games) [1,56], empathetic and friendly communication styles [1,56-58], a nonjudgmental tone [3,48], therapeutic tools like mindfulness and bibliotherapy [58-60], and personalized responses [57].

While existing literature sheds light on experiences with mental health technologies, less is known about how such experiences contribute to therapeutic relationships. A recent qualitative study of the DTA examined fully automated apps (including meditation tools and mental health chatbots) and identified five dimensions shaping connection: emotional resonance, perceived safety, goal alignment, on-demand availability, and the user’s own role in sustaining engagement [18]. These dimensions apply across non–dialog-based apps and provide a general jumping-off point to focus on how conversational interactions affect relational development. This study builds on this work by focusing specifically on mental health chatbots capable of two-way communication. By examining how conversation style, preferences, and interaction design influence DTA over time, we extend the understanding of relational mechanisms in more conversational, dialogue-based systems.

### Research Question

This research seeks to deepen our understanding of the relationship that forms between humans and digital mental health chatbots. Specifically, it aims to explore the nature of these bonds and how individuals perceive them. To achieve this, the study gathers participants’ perspectives on their interactions with mental health chatbots, examining whether they subjectively feel a sense of relationship with the chatbot. Additionally, it identifies key themes that shape and influence the development of these connections. The research question leading this study is the following: What are the themes in people’s perceptions about developing relationships with mental health chatbots? By addressing this question, the study aims to reveal the elements that foster human-chatbot relationships in the context of mental health care.

## Methods

### Chatbot Selection

Two well-established mental health apps, namely Woebot and Wysa, were selected for investigation. Both chatbots, based on cognitive behavioral therapy, an evidence-based approach, provide guided self-help treatments that typically range from 2 to 12 weeks [61-64]. Woebot has proven effective in alleviating symptoms of anxiety and depression while supporting overall well-being [1,3]. Wysa is a responsive chatbot with empathetic support and encourages users to delve deeper into their feelings [17]. Wysa also demonstrated efficacy in promoting well-being, exhibiting high engagement, and garnering positive acceptability [17,44,45]. This study used the publicly available versions of Woebot and Wysa between September 2022 and May 2023. At the time of this study, both Woebot and Wysa operated mainly with predefined decision pathways and natural-language classifiers, rather than open-ended generative AI [1,3,17].

As previous studies have shown, a sense of control plays a key role in shaping patients’ relationships with therapists [31-34]. Similarly, Wysa and Woebot represent two distinct approaches to conversation control. Wysa, with its free-text input and minimal guidance, allows users greater control over topics. In contrast, Woebot, with its predefined reply options and structured conversation flow, provides users with less control, potentially influencing how they perceive their relationship with the chatbot. Wysa adopts a more passive role, engaging minimally and waiting for users to initiate conversations. It primarily listens, encourages users to express themselves, and refrains from interrupting, steering discussions, or offering unsolicited advice. In contrast, Woebot takes a more proactive approach, frequently initiating conversations and greetings. It introduces topics, guides discussions in its own direction, provides advice, and shares more about itself. These differences resemble two distinct counseling styles.

**Figure 1:**
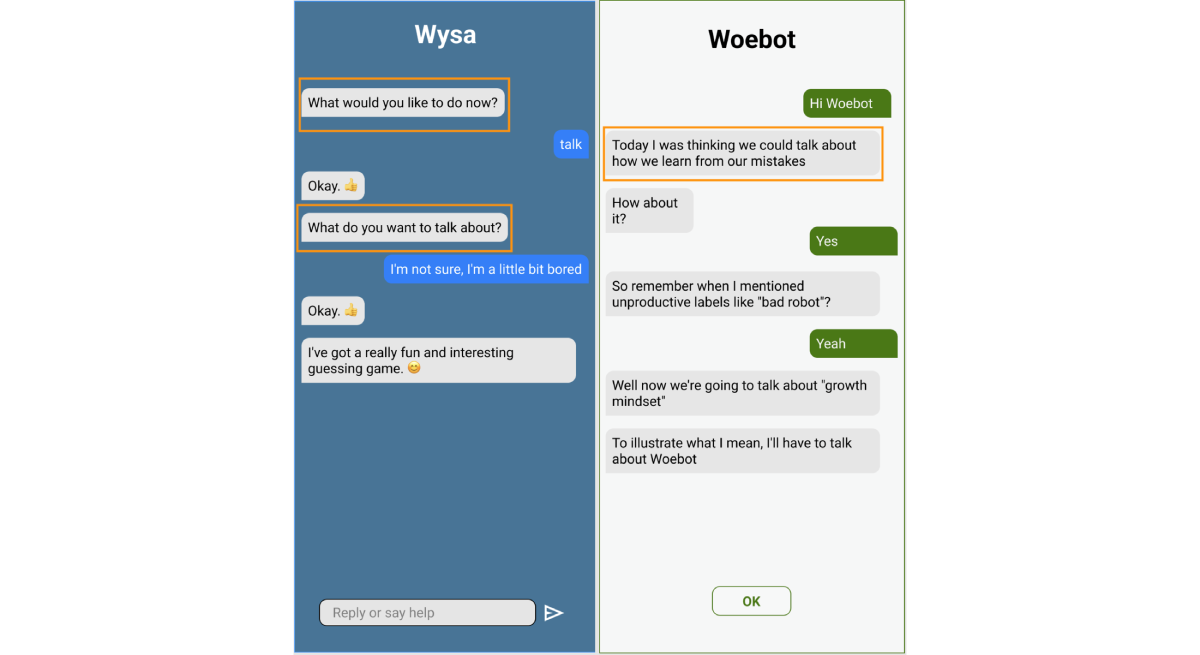
Comparison of chatbot communication styles: a more passive tone (left, Wysa) versus a more directive tone (right, Woebot). Yellow callouts highlight contrasting conversational roles—Wysa invites users to choose what to do and fosters self-expression, while Woebot guides users through predefined topics with a coaching tone. Simplified interface depictions by authors.

**Figure 2:**
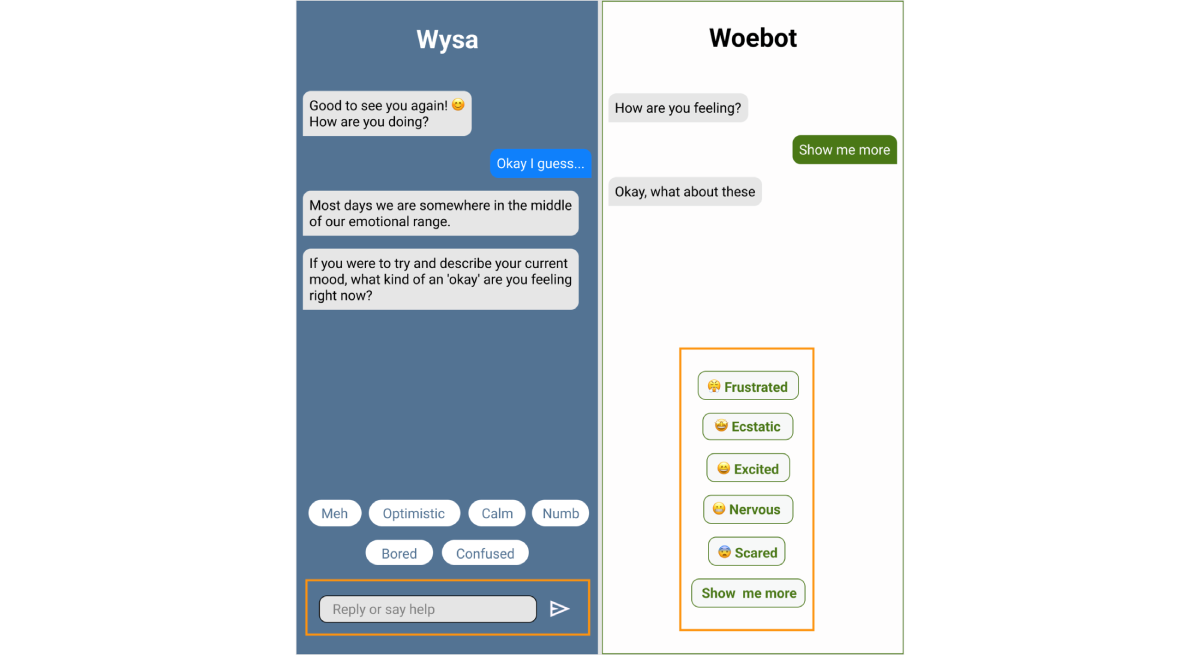
Comparison of chatbot input options: flexible free-text choices (left, Wysa) versus predefined responses (right, Woebot). Yellow callouts highlight interface differences—Wysa supports open-ended input alongside multiple-choice prompts, whereas Woebot requires users to select from fixed options, limiting conversational flexibility. Simplified interface depictions by authors.

As shown in Figure 1, when users engage with Wysa, the chatbot prompts them to decide what they want to do, encouraging them to take the lead in conversations. In contrast, Woebot typically provides predefined topics and directs users to follow its guidance. Wysa adopts the role of a listener, fostering self-expression, while Woebot acts more like a coach, leading users through structured interactions. Additionally, Wysa offers a free-text input field with fewer prompts, allowing for open-ended conversations. Woebot, on the other hand, relies on structured responses, requiring users to select from preset options. As seen in Figure 2, Wysa provides both multiple-choice options and an open-text box, creating a more flexible conversational experience. In contrast, Woebot limits responses to predefined options—when users cannot find a suitable response, they must request additional options until they find a close match or exit the conversation. This demonstrates Woebot’s closed-ended conversation style. Beyond conversation style, the chatbots also differ in their design. Visually, Wysa incorporates cute illustrations, while Woebot uses more humorous and lighthearted visuals. Wysa embeds videos and resources directly within the app, allowing users to access them seamlessly, whereas Woebot provides external links that require users to open a separate web page. Further design differences exist in areas such as how user reports and questionnaires are presented. These variations offer a variety of experiences and relationship dynamics, enabling analysis of features that influence bond formation.

### Study Design

#### Overview

This study used a 4-week diary study methodology, a short-term longitudinal qualitative approach designed to capture participants’ lived experiences in real-world contexts. This method is particularly suitable for studying human-chatbot interaction in naturalistic settings, allowing for rich, ecologically valid data collection on emotions, behaviors, and reflections over time [65,66]. Diary studies can involve various forms of data collection, including screenshots, voice memos, and participant annotations [66-69]. We adopted a similar approach, asking participants to document moments of emotional significance—such as engagement, disconnection, or expressiveness—while using the chatbot.

Participants engaged with two mental health chatbots—Woebot and Wysa—each for a 2-week period. Given that prior research has shown meaningful mental health improvements can occur within 2 weeks of chatbot use [1], this duration was selected as sufficient to observe the emergence of relational dynamics. Participants were not forced to engage daily but were encouraged to interact regularly based on their preferences and needs. They were also invited to capture screenshots and brief notes during emotionally salient moments.

To assess changes in emotional well-being, the World Health Organization—Five Well-Being Index (WHO-5) was administered at baseline and biweekly. This tool measures mood, vitality, interest, and general life satisfaction on a scale from 0 to 100, with a threshold of ≤50 indicating possible depression [70,71]. In this study, if scores fell below this threshold, participants were provided with information about support resources, such as University Health and Counseling services. Weekly semistructured interviews were conducted to collect qualitative data on participants’ experiences. These interviews provided opportunities to discuss emotional reactions, interpret chatbot behaviors, and reflect on relational developments.

#### Participant Recruitment and Eligibility

Participants were recruited through university-wide advertisements and mailing lists. Eligibility criteria included (1) current university enrollment, (2) aged 16 years or older, (3) ability to understand and respond in English during chatbot interactions and interviews, (4) interest in interacting with mental health chatbots, and (5) willingness to complete weekly interviews over 4 weeks. No prior relationship was established between the researcher and participants before the study began. We limited participation to currently enrolled students to streamline recruitment and ensure alignment with ethics protocols for low-risk university populations. In addition, access to support services (eg, University Health and Counseling) was contingent on student enrollment, enabling us to provide participants with appropriate resources if needed.

Prior to participation, eligible individuals attended a web-based onboarding session that outlined the study’s aims, procedures, confidentiality measures, and participants’ rights. Written informed consent was obtained after reviewing an information sheet. Participation was voluntary, and individuals could withdraw at any time without penalty. Because the lead researcher is bilingual in Mandarin and English, some bilingual participants were offered the option to conduct their interviews in Mandarin to facilitate more comfortable and nuanced verbal expression when reflecting on emotionally sensitive topics. This arrangement was intended to support deeper insight during interviews. Nonetheless, all participants met the proficiency requirements for university study in English as required for their enrollment, and thus met the study’s eligibility criteria of sufficient English proficiency to engage with the chatbots and participate in the study.

Initially, 34 individuals enrolled in the study. After excluding 8 participants due to withdrawal or incomplete WHO-5 data, the final sample included 26 participants (15 women and 11 men), aged 17 to 35 years (mean age 23, SD 4.66 y). Participants were recruited from computer science (n=16) and psychology (n=10) disciplines and received NZ $50 (US $30) upon completing the 4-week protocol, which included weekly surveys and interviews.

**Figure 3:**
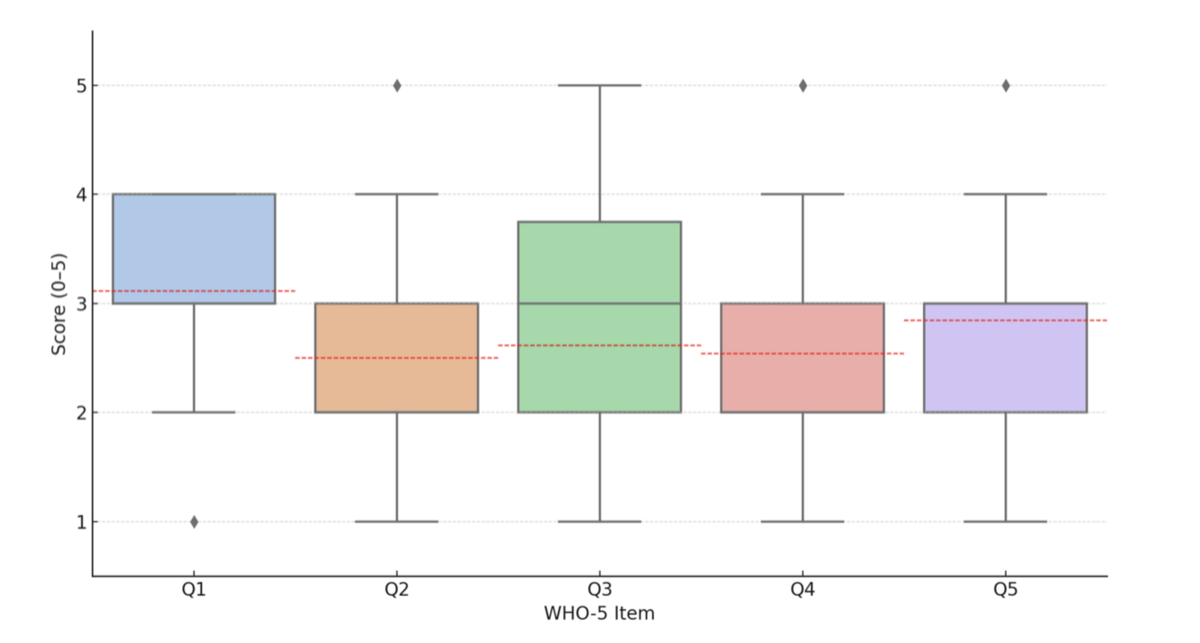
Item-level distribution of WHO-5 responses across five questions. WHO-5: World Health Organization—Five Well-Being Index.

At baseline, participants’ WHO-5 scores ranged from 32 to 80 (mean 54.77, SD 13.42). In total, 12 individuals scored below the clinical threshold of 50, which is indicative of poor well-being. Table 1 displays the total WHO-5 score distribution, while Figure 3 shows item-level response patterns across the 5 questions (rated on a 5-point Likert scale from 1=none of the time to 5=all of the time). Red dashed lines indicate the mean response for each item. Although most participants scored above the threshold, the item-level data suggest generally moderate emotional well-being.

Interview data further revealed that participants across the full range of WHO-5 scores expressed a strong interest in improving their mental health and exploring digital support tools, despite most having no prior experience with mental health apps.

**Table 1:** WHO-5^a^ Well-Being Index total scores (0-100): frequency distribution.

WHO-5 score	Participants (N=26), n (%)
32	1 (4)
36	2 (8)
40	2 (8)
44	1 (4)
48	6 (23)
52	2 (8)
56	1 (4)
60	3 (12)
64	2 (8)
68	2 (8)
72	1 (4)
76	2 (8)
80	1 (4)

^a^WHO-5: World Health Organization—Five Well-Being Index.

#### Study Procedure

**Figure 4:**
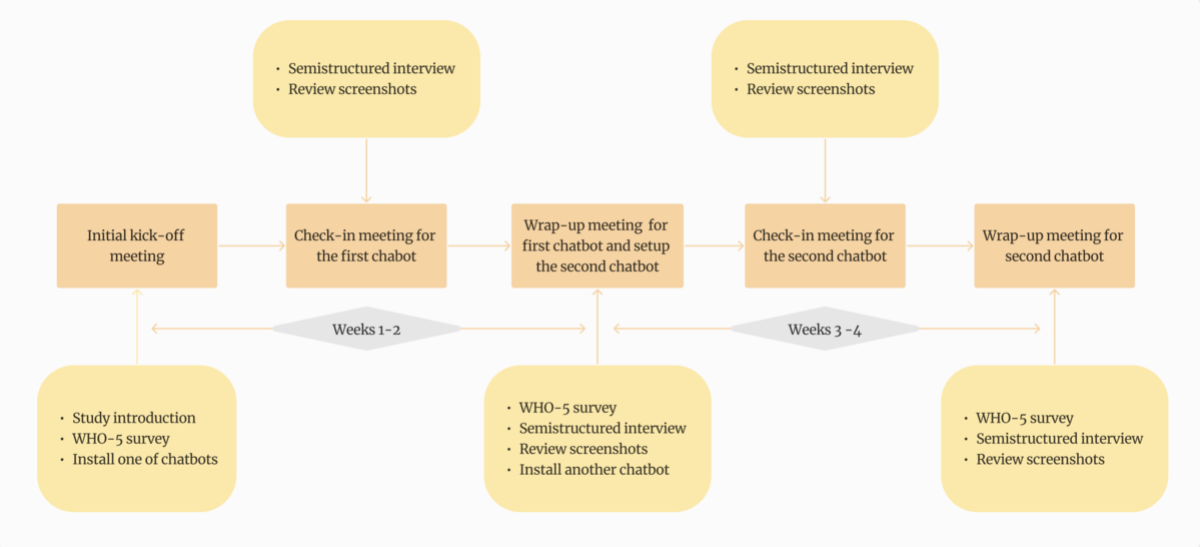
Study procedure. WHO-5: World Health Organization—Five Well-Being Index.

A visual overview of the study timeline and components—including chatbot use periods, weekly interviews, and WHO-5 assessments—is presented in Figure 4. The study took place over 4 weeks. During an initial onboarding session, researchers also guided participants through chatbot installation and conducted short test interactions (1-2 min) to ensure usability. Participants were randomly assigned to two groups in a crossover design: Group A used Woebot for the first two weeks, followed by Wysa, while Group B followed the reverse order. This design controlled for potential order effects.

Participants were encouraged to use each chatbot for 5-10 minutes per day or to complete 1-2 conversations per session, where one conversation referred to a person-initiated interaction that continued until the chatbot completed its suggested activity or dialog flow. They were asked to take screenshots of interactions that felt emotionally relevant and to annotate them with reflections or reactions. They were encouraged to blur any sensitive information to maintain privacy and were not required to capture a specific number of screenshots. All participation was individual, with no interaction between participants.

At the end of each week, participants joined a 30- to 90-minute web-based Zoom (Zoom Video Communications, Inc) interview, conducted in English or Mandarin based on preference. These semistructured interviews explored their weekly experiences with the chatbot. Participants also reviewed their screenshots and reflected on why they had captured those particular moments. The number and content of interview questions varied depending on how many weeks they had used the chatbots. Interview content was audio-recorded and later transcribed. Participants completed the WHO-5 survey at Weeks 0, 2, and 4 to monitor changes in well-being. The interview guide is provided in Multimedia Appendix 1.

### Ethical Considerations

This study was approved by the University of Auckland Human Participants Ethics Committee (24040). All participants provided informed consent prior to participation, and they were informed that they could withdraw from the study at any time without penalty. To ensure privacy, no personally identifiable information was collected, and all data were anonymized before analysis. Participants received NZ $50 (US $30) as compensation for their time. All procedures complied with institutional regulations and the principles outlined in the Declaration of Helsinki.

### Data Analysis

We used reflexive thematic analysis as outlined by Braun and Clarke [72,73] to explore how participants formed relationships with mental health chatbots over time. This approach acknowledges that meaning is coconstructed between researchers and participants. Rather than seeking coding consensus or interrater reliability, it prioritizes interpretive depth, reflexive engagement, and theoretically informed reading of participants’ accounts. In line with this approach, our analysis was primarily inductive and semantically grounded in participants’ explicit descriptions rather than pre-existing theory or latent interpretation. This study followed the COREQ (Consolidated Criteria for Reporting Qualitative Research) guidelines to enhance transparency and reporting rigor (Multimedia Appendix 2) [74].

The dataset included weekly semistructured interviews and annotated chatbot screenshots from 26 participants, yielding over 110 hours of audio data and more than 1500 images. We observed data saturation [75] after analyzing material from approximately 21 participants, when no new themes or conceptually novel insights emerged, supporting the sufficiency of the sample size and analytic scope.

All interviews were conducted by a bilingual doctoral researcher in computer science, with a focus on HCI and digital mental health. Two additional researchers—one with a background in psychology and the other in computer science—each joined approximately half of the sessions to support data collection. All researchers took detailed field notes and memos during and after interviews to support reflexive analysis. Interview protocols were adapted over time: during the first 2 weeks, questions focused on participants’ immediate experiences using the chatbots (eg, “How did you feel using the chatbot this week?”). In weeks 3 and 4, the same core prompts were retained to support continuity, while additional questions were introduced to explore emerging relational dynamics and invite comparisons between the two chatbots. In the final interview sessions, themes that had emerged earlier in the study were lightly introduced as flexible prompts when participants did not raise them spontaneously. This approach aimed to support reflection without narrowing the conversation, allowing participants to respond freely based on their own perspectives. Screenshots submitted by participants were used as contextual prompts to elicit deeper reflections on their interactions. These visual records were particularly helpful in surfacing moments of connection, misunderstanding, or disengagement that might not have been captured through verbal recall alone. Field notes taken during and after interviews provided additional contextual cues and helped interpret participants’ reactions to specific exchanges, particularly when screenshots conveyed ambiguous or emotionally nuanced content. This integration of multiple data sources aligns with reflexive thematic analysis, which emphasizes researcher subjectivity and iterative interpretation grounded in participants’ evolving accounts.

In addition to developing experiential themes inductively, we conducted a complementary deductive content analysis [76] to classify how participants described their overall relationship with the chatbots: Bond, Light Bond, and No Bond. This analysis drew on responses to recurring interview questions, such as “How would you describe the relationship between you and this chatbot?” and how participants described their overall relational experience, often in the context of emotional support, a feeling of being collaborative, and perceived understanding. Based on participants’ accounts, we categorized participant-chatbot relationships into one of the three categories of relational experience, typically based on the consistent presence of these elements. Ambiguous cases were reviewed collaboratively by the research team to ensure consistency.

Thematic development proceeded iteratively and reflexively over 5 months. The analysis team included the three researchers involved in data collection, as well as two senior researchers with expertise in digital mental health and HCI. The lead researcher began by identifying early patterns of meaning across transcripts and screenshot narratives. The other two researchers independently engaged with selected interview sessions and screenshots to provide alternative readings, challenge assumptions, and bring in varied disciplinary perspectives. Themes were initially explored separately for interview and screenshot data to preserve the nuances of each modality. Across the team, this process resulted in approximately 20 initial themes. In team discussions, we refined these themes by merging similar ideas, removing those that were unrelated to relationship development, and developing clearer definitions. These meetings also allowed us to reflect on how our own backgrounds and assumptions shaped the way we interpreted the data. Through this process, we arrived at a final thematic structure that all team members agreed captured both the conceptual clarity and the range of participant experiences observed in the data.

Although most participants spoke primarily in English, several incorporated Chinese in their annotations or interviews. These excerpts were translated by the bilingual lead researcher and reviewed for cultural nuance and accuracy by a second bilingual team member. Minor ambiguities in culturally specific expressions were discussed and resolved. Reflexivity remained integral throughout: the core team, comprising researchers from Chinese, bicultural, and North American backgrounds, regularly reflected on how their own positionalities shaped theme development, especially in interpreting relational norms and emotional expression in cross-cultural contexts of human-chatbot interaction.

## Results

### Overview of Themes and Bond Formation

This study identifies six major themes that shape and influence the relationship between humans and mental health chatbots. We describe these along with illustrative quotes.

Among participants who scored below the WHO-5 threshold (n=12), 7 reported forming either a strong or light emotional bond with at least one chatbot. Similarly, 11 of the 14 participants who scored above the threshold also described such relational experiences. These findings indicate that the capacity to perceive a bond with a mental health chatbot does not appear to be limited by one’s mental health status at the time of participation. Thematic analysis of interviews revealed that participants from both groups, regardless of WHO-5 score, discussed similar types of relational experiences. All six themes drew responses from participants across both WHO-5 groups (at times for only a single chatbot), as presented in Table 2, reflecting that these relational aspects were widely encountered, even if experienced and interpreted in varied ways.

**Table 2:** Participant distribution across six themes by WHO-5^a^ group (<50 vs ≥50)^b^.

Theme	Below the WHO-5 threshold, n (%)	Participant IDs below threshold	Above the WHO-5 threshold, n (%)	Participant IDs above threshold
Theme 1	11 (92)	5, 8, 10, 13, 14, 16, 20, 23, 24, 25, 26	13 (93)	2, 3, 4, 6, 7, 9, 11, 12, 17, 18, 19, 21, 22
Theme 2	12 (100)	5, 8, 10, 13, 14, 15, 16, 20, 23, 24, 25, 26	14 (100)	1, 2, 3, 4, 6, 7, 9, 11, 12, 17, 18, 19, 21, 22
Theme 3	11 (92)	8, 10, 13, 14, 15, 16, 20, 23, 24, 25, 26	13 (93)	1, 2, 3, 4, 6, 7, 9, 11, 12, 18, 19, 21, 22
Theme 4	10 (83)	5, 8, 10, 13, 15, 16, 20, 23, 24, 26	13 (93)	1, 2, 3, 4, 6, 7, 9, 11, 12, 17, 18, 21, 22
Theme 5	11 (92)	5, 8, 10, 13, 15, 16, 20, 23, 24, 25, 26	11 (79)	2, 3, 4, 6, 7, 9, 11, 12, 18, 21, 22
Theme 6	9 (75)	10, 13, 14, 15, 16, 23, 24, 25, 26	9 (64)	2, 7, 11, 12, 17, 18, 19, 21, 22

^a^WHO-5: World Health Organization—Five Well-Being Index.

^b^Percentages reflect the proportion of participants within each WHO-5 category who contributed any quote coded under the corresponding theme. Because some contributions were brief, tangential, or neutral—and some reflect experiences with only one chatbot—100% does not indicate full or consistent endorsement across participants.

Based on participants’ interview data, we identified three categories of relational experience: Bond (B), Light Bond (LB), and No Bond (NB). Participants categorized as Bond (B) described forming a clear emotional connection and bonding with one chatbot, often likening it to “talking to a friend” or “talking to a coach or therapist.” For instance, Participant #2 shared, “Woebot made me feel like it was a friend who was encouraging you to complete a task as you are so close.” Those categorized as No Bond (NB) did not perceive any relationship with the chatbots, describing them as “just a tool,” “like a stranger,” or “acquaintance,” or simply stating there was “no relationship.” A subset of participants reported a more neutral or tentative connection, categorized as Light Bond (LB), often describing the chatbot as “a friend but not too close,” “a long-distance coach,” or expressed that they felt a little bit bonded with the chatbot. For example, Participant #1 noted, “I think after stopping using Woebot, I actually kind of missed it. So I definitely felt a bit of a bond with Woebot.”

To present participants’ perceived bonding levels with the two chatbots, we use a standardized shorthand notation in the format N#BondLevel–WB|BondLevel–WY (eg, N11LB–WB|NB–WY), where bond levels are abbreviated as B (Bond), LB (Light Bond), and NB (No Bond). The app order is fixed as WB|WY, referring to Woebot and Wysa, respectively. For readability, the simplified notation N#LB|NB is used throughout the paper, with the WB|WY order assumed. For example, N11LB|NB indicates that Participant 11 reported a Light Bond with Woebot and No Bond with Wysa. In total, 18 participants reported perceiving a bond or light bond with at least one chatbot, while 8 participants did not perceive any bond with either chatbot (NB). In the following themes, we begin with findings related to Woebot, followed by those on Wysa. To illustrate how participants experienced emotional connection and relationship development with the chatbots, we include participant identifiers (N#LB|NB) alongside representative quotes from those who reported varying levels of bonding. Table 3 further summarizes the types of bonds participants reported, grouped by their WHO-5 well-being scores. Participants were categorized as either above or below the threshold score of 50. As shown, both groups included individuals who experienced a bond, a light bond, or no bond with the chatbots. This distribution indicates that the ability to perceive a relational connection with a mental health chatbot is not strictly dependent on one’s current level of psychological well-being.

**Table 3:** Distribution of bond types among participants categorized by WHO-5^a^ scores.

WHO-5 group	Bond type distribution (N)	Participant IDs
Below threshold	Woebot: B (3), LB (3), NB (6) Wysa: B (3), LB (1), NB (8)	N5LB|NB, N8B|NB, N10B|NB, N13NB|NB, N14NB|NB, N15B|B, N16LB|NB, N20NB|NB, N23NB|NB, N24NB|LB, N25NB|NB, N26LB|B
Above threshold	Woebot: B (6), LB (6), NB (2) Wysa: B (7), LB (0), NB (7)	N1LB|NB, N2B|B, N3LB|NB, N4LB|B, N6LB|B, N7LB|NB, N9NB|NB, N11LB|NB, N12B|B, N17NB|NB, N18NB|NB, N19B|B, N21B|B, N22B|NB

^a^WHO-5: World Health Organization—Five Well-Being Index.

### Theme 1: The Desire to Lead or Be Led in Conversation

#### Overview

Participants had varying preferences for managing conversations with mental health chatbots, influenced by their need for control or their desire to cede control. Participants reported that their control over interactions impacted their connection and relationship quality. Some preferred leading conversations by themselves, while others appreciated chatbot-led discussions that introduced new topics or timely interruptions, which some found helpful for keeping the conversation moving. Participants suggested that a dynamic approach, where the chatbot adapts to a person’s needs, would provide a better balance of autonomy and guidance.

#### Person-Led Conversations

Some participants preferred to lead conversations themselves, finding it more engaging and tailored to their needs. But Woebot does not support a person-led conversational style, which led some participants to feel disconnected and constrained during their interactions. N6LB|B described feeling disconnected when they were unable to redirect the conversation, highlighting how Woebot’s rigid flow limited their sense of control—despite the exchange initially seeming open-ended:

It got really boring and mundane. I didn’t want to talk about(...), and now I have to talk about it for ages. I felt very disregarded because I didn’t have a choice.

N6LB|B

Participants praised Wysa for offering them the freedom and control to guide the conversation, which contributed to the development of a bond. For instance, one participant explained their perception of a light bond with Wysa:

I like to start the chat by myself because it’s like a tool for my emotions. I need to share and talk about more.

N24NB|LB

Moreover, this feeling was echoed by another participant, who shared a screenshot capturing a moment of perceived connection during the interaction, even though they ultimately did not feel a sustained bond with Wysa:

It can let me take charge of the conversation by saying what I want to say.

N23NB|NB

Additionally, N23NB|NB further reflected that the sense of bond was disrupted by interactions that felt impersonal, overly scripted, and overly focused on promoting services, ultimately breaking the illusion of a genuine connection.

However, Wysa’s conversation style sometimes posed challenges for those who struggled to initiate or direct conversations. A participant shared:

I didn’t want to start chatting and didn’t have much to share. It left me feeling frustrated.

N5LB|NB

#### Chatbot-Led Conversations

Some participants appreciated when chatbots took the initiative in conversations, especially when they are in a bad mood or need guidance. They valued Woebot’s ability to lead discussions and introduce new topics. N8B|NB built the bond with Woebot and remarked:

Even though Woebot usually takes charge of the chat most of the time, I can stay very focused during our chat.

This indicates that a chatbot-led approach can be effective for people who benefit from a structured dialogue. N11LB|NB further highlighted the benefits of this approach by stating the following statement. This proactive topic introduction helps maintain engagement and stimulates ongoing conversation:

It shares new topics every day, I feel curious and want to keep chatting with it.

N11LB|NB

In contrast, Woebot’s tendency to drive the topics of the conversation led to frustration. One participant expressed concern with Woebot’s lack of conversational control, said:

It brings up a topic and then just keeps talking to itself. It gives you stuff to think about, but you can’t really have a chat with it—you’re just sitting there taking it all in.

N9NB|NB

These responses highlight a gap between expectations and the chatbot’s style in managing the dialogue, underscoring the need for more balance in conversational control.

On the other hand, Wysa does not support a chatbot-led conversational style. Participants who preferred Woebot’s more structured and directive approach compared it to Wysa’s, noting that they appreciated having clearer guidance and direction in the interaction, as shared by one participant:

Woebot usually starts with a topic, which makes it easier for me to follow and want to open up. I kinda like how Woebot starts the conversation—feels more natural.

N12B|B

#### Dynamic Control in the Conversation

Participants also expressed a preference for dynamic conversational control, where the chatbot adapts to their needs. One participant explained:

If I have a particular problem, then leading the conversation can be quite a lot more useful. If I don’t really have that or if I don’t understand my own problems, then having it lead the conversation can be quite helpful.

N22B|NB

N11LB|NB echoed this view, describing how their preference shifted based on emotional state:

If I was feeling down that day, I wanted to start the chat myself. But on a normal day, I preferred the chatbot to suggest the topic.

This feedback indicates a desire for flexibility, where the chatbot can switch between leading and following based on the person’s needs. This dynamic approach allows for a more tailored interaction, potentially enhancing the relationship. Other participants, including N18NB|NB and N14NB|NB, also supported this flexible control, suggesting that a responsive chatbot can better meet their needs and improve their relationship with chatbots.

### Theme 2: The Match Between the Ability and Desire to Self-Express: Open-Ended Style (Free-Text) or Closed-Ended Style (Provided Prompts)

#### Overview

Participant communication preferences, such as open-ended versus closed-ended conversation style, showed influence on their relationship with chatbots. Woebot uses prompts, such as a handful of one-word mood options that the user can select in response to the question “How are you feeling today?” which provides a structured conversation flow. Wysa offers free-text input, which allows users to open up and talk about their own topics.

#### Preference for Free-Text Input

Many participants valued free-text input for its ability to facilitate self-expression, which they felt strengthened their connection with the chatbot. In some instances where Woebot allowed free-text input, participants often recorded these moments and described feeling a sense of connection with the chatbot during these less restricted exchanges, suggesting that even brief opportunities for free-text input contributed to perceived bonding. For instance, one participant echoed this sentiment, stating:

Woebot shares its own stuff (...) When I can type my own thoughts, it makes me want to open up more. [explaining the moment they felt a bond].

N11LB|NB

Wysa’s support for free-text input was frequently cited by participants as a key interaction contributing to moments of perceived bonding. Many participants emphasized this during interviews, noting that the ability to express themselves in their own words made interactions feel more personal and meaningful. For instance, N20NB|NB recalled a moment of connection, as illustrated in the quote below, even though they ultimately did not feel a sustained bond with Wysa. This highlights how open-text input can enhance connection and support the development of relational closeness with the chatbot:

Typing whatever I want, it is kind of like chatting with a friend.

However, free-text input did not always suit every situation. N11LB|NB also shared why they did not feel a bond with Wysa, saying:

When I chat with it at night, I’m usually exhausted and don’t want to think too hard. So, I’d rather just pick from options than type out my own stuff.

This view was consistent with N18NB|NB’s opinion, noting that while free-texting can enhance bond, it may be less practical during times of fatigue.

#### Preference for Predefined Options

While many participants favored free-text input, predefined options were useful in some circumstances. Predefined options helped participants initiate conversations more easily and maintained the flow of interaction. One participant, who did not build a relationship with Woebot but shared the moment when he felt the bond with it, stated:

I think the options are really easy to use. I actually prefer using them ... it feels relaxing and there’s no pressure. I don’t have to come up with what to say, and it’s easy to just go with the flow and move on to the next topic.

N13NB|NB

This view was echoed by N16LB|NB, who also appreciated the convenience of predefined choices. Participants who formed connections with Woebot valued platforms that adapted to their preferences. As N12B|B shared:

I’m not super expressive, so if the options don’t match exactly how I feel, I’m fine picking something close enough. It doesn’t really bother me.

On the flip side, limited options sometimes led to frustration, as one participant expressed:

It gives me prompts instead of actual input. It feels like I’m pressing buttons instead of typing, so it kind of takes away from the experience. I’m disappointed.

N23NB|NB

Regardless of their preferred expression methods, all participants emphasized the importance of chatbots accurately understanding their input to maintain engagement. Frustration arose when chatbots failed to comprehend free-text input, leading to a decrease in motivation to express themselves. As N1LB|NB explained why they lost the bond with Wysa:

It’s obviously not interpreting correctly what I’m typing in. So it’s kind of pointless.

### Theme 3: Expectations for Caring and Nurturing From the Chatbot

#### Overview

Participants expressed strong expectations that chatbots could act as emotionally supportive figures, providing comfort, empathy, and reassurance. This theme focuses on the emotional depth and relational quality participants sought in chatbot interactions—expecting to feel heard, comforted, and emotionally understood, much like with a caring human companion.

#### Fostering Bond Through Empathy and Emotional Depth

Participants sought attachment and closeness with mental health chatbots, emphasizing the need for emotional understanding and empathy. They believed that for chatbots to provide effective emotional support, demonstrating empathy was essential, as participants expected varying levels of understanding and kindness.

First, participants valued the basic comfort and care provided by chatbots, especially during moments of distress. Acts of reassurance, concern, and empathy—such as attentive listening and validating emotions—were key in strengthening the human-chatbot bond. Both Woebot and Wysa demonstrated these supportive behaviors, which contributed to building emotional rapport. This sense of comfort was reflected by one participant, who described a subtle feeling of connection with Woebot:

I felt like I was being looked after by Woebot. It seemed to care about me. I felt relieved knowing it’s okay to do nothing. It showed caring and empathy. I felt a little bit of a bond, like with a friend.

N8B|NB

A similar feeling was expressed by participants who interacted with Wysa. For instance, another participant noted:

When it said, “I heard you, and noted at all.” I felt (...) it was actually listening, not just taking a bunch of data from what I was saying.

N15B|B

This sense of being genuinely heard was also echoed by N2B|B and N21B*|*B, who described the responses as attentive and emotionally validating.

Second, participants expressed a desire for emotional support that went beyond surface-level engagement. They wanted chatbots not only to display empathy but also to engage in meaningful dialog that explored deeper emotional issues. This kind of support involved thoughtful prompts, reflections, and a willingness to address the root causes of distress. Wysa, in particular, was noted for facilitating such depth by encouraging participants to open up. As a participant shared:

The only difference is when they ask me about deeper feelings or emotions, I open up more and am more exposed.

N21B|B

However, this pursuit of emotional depth was a double-edged sword. When chatbots probed too directly or failed to convey sensitivity, participants could feel uncomfortable or even overwhelmed. As N9NB|NB reflected:

I felt uncomfortable because it kept asking about my problems.

#### Providing Consistent Emotional Support

To foster lasting emotional bonds, chatbots needed to strike a delicate balance between being empathetic listeners and offering practical guidance. Participants emphasized that while comfort and emotional support were crucial, they also appreciated actionable suggestions—so long as these were delivered with empathy. This balance, however, was not always easy to maintain. When the chatbot’s tone or approach shifted too abruptly, it could disrupt the emotional connection and reduce the sense of being understood. As one participant, who felt a light bond with Woebot, reflected:

I was surprised by how comforting and empathetic it could be. But it needs to be consistent. One day it was really comforting, but the next day it just felt like it was teaching and didn’t bring empathy.

N26LB|B

A similar sentiment was echoed by Wysa users. When chatbots failed to demonstrate empathy, participants described feeling disconnected or even dismissed. Without emotional resonance, the interaction risked becoming cold or mechanical. For instance, N12B|B shared a moment when the bond with Wysa broke down:

I told it I was in a bad mood and hoped it would continue to give me some comfort and suggest things I could try. But it just sent me a bunch of hotlines.

### Theme 4: Relevance of the Chatbots’ Psychoeducation and Joint Activities

#### Overview

Participants reported that interactions needed to feel regularly “valuable,” including engaging through diverse types of content such as text-based reflections, digital exercises, and professional psychoeducational resources. Repetitive activities and unrefined content were found to disrupt the process of building a relationship.

#### Sustained Use and Meaningful Engaging Activities

Participants emphasized that regular daily use, offered by both chatbots, contributed positively to relationship-building. Routines, predictability, and consistent check-ins helped establish a sense of familiarity and structure, which was reassuring over time, as one participant described the impact of these regular interactions with Woebot:

The bond is developing well [by using daily check-ins], but it’s not strong enough.

N8B|NB

After participants established familiarity through regular use of chatbots, their relationships with the chatbots were further strengthened by joint activities, games, and exercises. Engaging activities like goal-setting tasks, breathing exercises, workouts, and meditation, offered by both chatbots, kept participants motivated and fostered a deeper connection, making interactions more beneficial and fulfilling, as one participant highlighted:

I really enjoyed a lot of activities. It felt friendly and was easy to navigate. I definitely felt engaged by the app. And it was quite helpful, supportive, and warm. And it made me feel empowered.

N24NB|LB

Moreover, collaborative exercises offered by both chatbots fostered a sense of bonding by creating shared experiences. These activities encouraged participants to reflect on positive aspects of their lives, helping to deepen their emotional connection with the chatbot and enhancing feelings of support and understanding. As N6LB|B described, this sense of “doing it together” contributed to feeling emotionally supported—a sentiment echoed by N15B|B, N8B|NB, and N22B|NB:

I could say there was a connection there because it was telling me that “we all” kind of do it as a team, instead of just me, and it was like helping me out, it was like “we’ll figure it out together.” So this one made me feel quite understood, supported.

#### Professional Advice

Both chatbots provide constructive suggestions and psychological insights that enhance support and bonding. By offering practical tips and professional knowledge in relatable terms, they foster connection and make interactions educational and supportive. Positive reinforcement and helpful guidance strengthen the bond between the person and the chatbot, as one participant shared:

It uses psychological terms, which is pretty cool. It helps people who might not know things like CBT and guides them to explore their thoughts and solve problems.

N21B|B

On the contrary, excessive and impersonal advice could undermine the supportive nature of the interaction. When chatbots overwhelmed participants with too much information or presented advice in a detached manner, participants reported feeling annoyed or disengaged. As N18NB|NB noted:

I was reading quite a bit [with it], providing quite a bit of information. It was quite annoying to read. Sometimes I just don’t want to.

Moreover, repetitive or generic responses, unpolished content, and overwhelming follow-ups also led to frustration and emotional disconnect, as N3LB|NB shared:

The chat keeps asking the same mood question every day, and if I pick “OK, I guess,” it just gives me the same reply every time. It gets kind of boring and makes me feel a bit lonely.

### Theme 5: Appreciation for Human-Like Colloquial Communication

Unlike Theme 3, which focuses on expectations for emotional empathy and support, and Theme 1, which centers on preferences for directing the flow of conversation, this theme highlights how colloquial communication styles and replies that demonstrated awareness of prior exchanges shaped participants’ sense of bond—by making interactions feel more natural, responsive, and attractive.

Participants largely appreciated chatbots that exhibited human-like qualities and demonstrated awareness of previous conversations, finding that these elements fostered connection. Participants emphasized the importance of a human-like colloquial communication style. The use of engaging features like emojis and shared stories created a sense of empathy and understanding. Conversely, scripted or irrelevant responses that lacked tailored understanding weakened the bond, making interactions feel impersonal and ineffective.

For example, some participants described feeling “noticed” when the chatbot remembered past exchanges or picked up on previously shared content. These follow-ups made the interaction feel more like a continuing relationship than a series of isolated conversations. As N12B|B shared—and a sentiment echoed by N10B|NB and N3LB|NB about Woebot:

It said that I’d said (…) before and suggested starting a new topic based on what I’d said. It made me feel like he remembered and cared about what I’d shared, which made me feel that our relationship was deepening.

Participants also valued when the chatbot’s language and emotional tone felt personally relevant and emotionally attuned, which enhanced their sense of being seen and understood. Moreover, when chatbots demonstrated the ability to recognize emotions and respond with empathy, sometimes even sharing similar experiences, participants felt genuinely understood, which helped deepen the connection. As N26LB|B explained, a key reason they formed a bond with Wysa was the chatbot’s replies that built on their earlier conversations:

[I felt the bond because] the replies that were given were tailored to my response.

Similarly, N8B|NB, who ultimately did not form a strong bond with Wysa, still recalled a moment of connection when the chatbot demonstrated emotional recognition:

It seemed to understand me and shared the exact same experience I had. It made me feel much more understood and closer.

Conversely, when chatbots delivered generic responses, interactions often felt impersonal and one-sided. This made the chatbot seem disconnected and uncaring, reducing its perceived bond, as one participant explained:

If it just talks about what it wants to say without considering my feelings, it feels like it doesn’t really understand me. The advice ends up being generic and not helpful for my specific situation, which makes it feel pretty pointless and perfunctory.

N9NB|NB

Finally, visuals or jokes that seemed irrelevant or overly scripted diminished the appeal, making interactions feel robotic. Some participants expressed frustration over mismatched tones, noting that these elements detracted from their overall experience.

### Theme 6: Valuing a Private and Nonjudgmental Conversation

Trust played a central role in building relationships with mental health chatbots, and this theme gathers evidence on how trust was established and the elements that could undermine it. Participants discussed how their trust in chatbots developed over time. Key elements for building trust included creating a safe, nonjudgmental environment, ensuring technological reliability, and protecting privacy.

Participants felt trust grew when chatbots provided a safe space where they could express their thoughts without fear of judgment. Both Woebot and Wysa received praise for fostering nonjudgmental environments, making participants feel comfortable sharing their emotions and personal issues. This sense of safety encouraged deeper engagement, as participants felt they could open up more freely. As N21B|B noted:

I trust it [Woebot]. It will not speak out about what I said.

I trust it [Wysa] a lot. It always gives the neutral side of ideas rather than personal ideas.

Additionally, privacy played a pivotal role in trust-building. Participants were more willing to share personal details when they believed their data were secure and confidential. As N25NB|NB highlighted the importance of privacy:

It seems like it’s [Woebot] very private, and I wouldn’t worry about any like information leakage.

Conversely, certain beliefs and behaviors eroded trust and hindered relationship development. Participants felt disconnected when chatbots appeared to have profit-driven motives or when they feared their information might be misused. Such concerns created barriers to open communication and reduced the chatbot’s effectiveness. As one participant explained the moment they lose the bond:

It makes you step back and realize, oh, you know, at the end of the day, this is just an app [Wysa] with a developer, and they need to get good ratings in the app store. It sort of breaks the natural flow of the conversation.

N2B|B

## Discussion

### Principal Findings

The study identified six key themes that impact the relationship between humans and mental health chatbots. First, preferences for leading or being led in conversations influenced the relationship with the chatbot, with some preferring more control while others value guidance. Second, the match between communication preferences—whether through free-text input or predefined options—and the chatbot’s style affected engagement and expression. Third, people sought emotional support that is empathetic and consistent. Fourth, participants valued meaningful, engaging activities and professional advice from chatbots, but repetitive or generic content hindered relationship development. Fifth, human-like communication, such as referencing past conversations, sharing stories, using colloquial language, and providing replies that demonstrated awareness of previous exchanges, supported relationships, while overly scripted or irrelevant content detracted. Finally, trust was essential for relationship building, with participants emphasizing the importance of privacy, nonjudgmental environments, and the chatbot’s perceived motives.

While each of the six themes reflects a distinct aspect of how people build relationships with mental health chatbots, we also observed meaningful overlaps between them. For example, emotional resonance often appeared alongside perceived human-likeness, suggesting that people were more likely to feel emotionally connected when the chatbot’s language and tone felt more natural or human. Likewise, conversational control was frequently mentioned together with expressiveness, indicating that people felt more in control when they could express themselves freely. These patterns suggest that relationship-building is shaped not by isolated factors, but by the interaction of multiple experiences. Future research could explore how these elements work together over time to influence how people relate to mental health chatbots.

### Comparisons With Prior Research

This study contributes to the DTA framework by examining interactions with mental health chatbots over a 4-week period, focusing on subjective experiences and the perceptions of relationship development with chatbots. Our findings contribute to the ongoing efforts to define and operationalize the DTA, as proposed in recent reviews, such as Malouin-Lachance et al [42]. While their work outlines key theoretical components of DTA, including goal alignment, task agreement, therapeutic bond, and user engagement, this study offers empirical insights into how these dimensions are experienced and interpreted during real-world interactions with mental health chatbots. It also helps clarify the scope of DTA, which holds potential for enhancing digital mental health interventions but remains underdefined [37,41]. Prior studies, including one that explored DTA across a range of mental health apps, have identified several factors impacting it [18]; our work validates their findings that beliefs about digital conversations being safe and nonjudgmental support relationship building, and that meaningful emotional interactions also play a key role. We extend DTA research by connecting it to work on two-way communication in social contexts, an important aspect in physician-patient relationships [31-34]. Additionally, we further the DTA framework with communication-related themes such as leadership in conversation topics, the impact of input modes on self-expression, and the role of colloquial communication style. Our theme on perceived effectiveness aligns with previous work on goal alignment, but also introduces the concept of “being” and “doing” together, linking our findings to those suggesting that diverse interactions foster relationship development [49].

Previous research on face-to-face therapy has shown that managing control dynamics in physician-patient interactions enhances communication and strengthens relationships in clinical settings [31-34]. Our first theme aligns with these insights, suggesting that similar dynamics are important in building therapeutic relationships with mental health chatbots. Previous studies recognized free-text input as enhancing user experience [48], but they did not link it to relationship development. Our findings do this and reveal that while free-text input contributes to positive experiences, this does not account for all communication needs—matching people’s preferred methods for self-expression is what helps to build relationships with chatbots.

Empathic interaction makes people feel cared for, understood, and connected when they receive emotional support [1,18,56-58]. Our research extends this with the specific types of emotional support that people expect, bringing up relevant aspects of previous conversations, consistent demonstrations of empathy, and balancing the role of empathetic listener and proactive coach. This study highlighted that the perceived effectiveness of the chatbot’s advice and proposed activities was linked to relationship development. This echoes previous findings within a social chatbot context [49], which mentioned chatbots’ ability to participate in a variety of interactions pushed the relationship toward attachment. Across the board, our participants appreciated human-like colloquial communication style and replies that demonstrated awareness of prior conversations, which aligns with past research [1,3,57]. In contrast to previous work, this study revealed that one specific aspect of a human-like colloquial style—recalling past conversations in future interactions—was particularly important for fostering a consistent connection and ongoing relationship development with chatbots.

While previous work found that specific therapeutic approaches and techniques [58-60] and diverse content [1,56] contributed to positive user experiences, these were not connected to mental health chatbot relationship development in this study. Our findings had a shifted focus toward perceptions of professionalism and diverse content for perceived effectiveness. We also found that creating a routine can help develop familiarity, which is in line with previous work showing that longer and more intense interactions with chatbots are associated with increased social connectedness [54]. Our research links routine interaction, increasing feelings of familiarity, with deepening relationships with chatbots.

Trust emerged as a fundamental aspect influencing initial willingness to use a chatbot, a point widely discussed in previous literature [3,46,48]. We also found that trust plays a foundational role in relationship development. Trust has been linked to a nonjudgmental environment, which has been linked to positive user experiences [3,48]. Our research further suggests that such an environment not only fosters trust but also encourages openness, allowing people to share their thoughts more freely. Privacy concerns were also highlighted in our findings as an important aspect of trust-building. The belief in a nonjudgmental and privacy-respecting environment fosters trust and a bond with mental health chatbots.

Although we did not directly apply structured instruments such as the WAI-S, D-WAI, or mARM, our categories (Bond, Light Bond, and No Bond) show meaningful alignment with these tools. For example, participants in the Bond group described experiences of collaboration, emotional support, and feeling understood—core features also emphasized in WAI and mARM frameworks. No Bond cases reflected disengagement and lack of emotional resonance, suggesting parallels with low alliance scores. Light Bond, meanwhile, captured more mixed experiences: participants described moments of connection or responsiveness, but without the consistency or emotional investment typically associated with full alliance. These cases reflect the kind of early-stage or partial alliance that structured tools may not always capture well. Importantly, our findings suggest that Light Bond may not simply represent a transitional stage toward a stronger alliance, but rather a distinct and meaningful relational state—emotionally real, yet fragile and easily disrupted. While low TA in traditional therapy is often associated with treatment dropout or reduced treatment efficacy [77], the Light Bond observed in chatbot contexts does not always lead to disconnection. Instead, some continued to use despite only a minimal sense of bond. This divergence highlights a key distinction between chatbot-mediated and human-delivered care: in digital contexts, even tenuous relational bonds may be sufficient to sustain engagement. These findings underscore the need for DTA frameworks to more explicitly account for the variability and fluidity of alliance formation in chatbot settings.

While many participants reported feeling bonded to the chatbot, they also emphasized that this sense of bond needed to be paired with therapeutic relevance. When interactions felt generic or failed to address their concerns, some questioned the chatbot’s purpose, suggesting they might prefer a general-purpose agent instead. This highlights an important distinction: general chatbots may offer companionship or entertainment, but mental health chatbots are expected to provide emotionally attuned support and help people work through personal challenges. In this context, participants’ expectations aligned more closely with the goals of a DTA—one that includes empathy, goal-setting, and responsive guidance. Without these elements, even warm or friendly interactions were seen as superficial, underscoring the need for design approaches that integrate both emotional connection and therapeutic intent.

Rather than relying on predefined rating scales, we show how alliance-related perceptions emerge through specific types of interaction, such as empathetic responses, collaborative tasks, and feeling remembered. These insights may inform future refinements of DTA theory and the development of hybrid measurement approaches that combine structured tools with narrative-based indicators of relational quality.

In addition, although our analysis did not specifically track changes over time, participants’ weekly reflections suggested that their sense of relationship with the chatbot often shifted. Some described initial curiosity that faded as conversations became repetitive, while others reported growing connection as they discovered useful or emotionally attuned features. These patterns highlight that DTA is dynamic—shaped by evolving expectations and experiences—underscoring the need for designs that support relationship development over time. Future longitudinal studies could further explore how such temporal dynamics influence relationship formation and sustainability.

Drawing from our findings, we propose three design suggestions to support early-stage DTA in mental health chatbots. First, chatbots should balance emotional support with actionable guidance, ensuring both are tailored to evolving emotional states and concerns. Participants valued empathy and advice, but only when responses felt relevant and attuned to their needs—generic replies often led to a lack of relational bond. Second, offering flexible input methods (eg, switching between free text and structured options) can accommodate communication preferences and enhance their sense of agency. Finally, continuity mattered: when chatbots recalled prior conversations or followed up on earlier topics, people felt seen and supported. Simple memory mechanisms that highlight relevant past exchanges may deepen trust and foster a more sustained relational connection. In addition, logging adaptive responses to user input—such as changes in tone or content—could support transparency and auditability, aligning with Article 22 of the European Union (EU) AI Act [78]. This design practice not only helps ensure accountability in automated decision-making but also builds trust by allowing users and regulators to understand how and why a chatbot responded in particular ways.

### Limitations and Future Work

This study has several limitations. First, while participants were encouraged to interact with the chatbots daily, some struggled to maintain consistent engagement due to busy schedules, declining interest, or perceived repetitiveness. This inconsistency highlights a challenge in sustaining real-world use and suggests that longer-term studies may be needed to capture how relationships with chatbots evolve over time. We therefore describe this as a short-term longitudinal study that primarily reflects early-stage dynamics of DTA formation. Second, during the study period, more advanced conversational agents—such as GPT—capable of understanding language in more flexible and adaptive ways, were beginning to attract public attention. Some participants drew informal comparisons between the chatbots they used and these emerging systems, highlighting a growing expectation for interactions that demonstrated awareness of prior exchanges. While generative AI systems promise more adaptive support, they may misinterpret distress or provide inaccurate guidance, raising concerns about safety, reliability, and clinical oversight. Regulatory frameworks such as the EU AI Act (Regulation [EU] 2023/1114) emphasize transparency, explainability, and safeguards against algorithmic bias—particularly under Article 22, which highlights the importance of traceability and meaningful explanation of automated decisions [78]. These principles could inform how memory and relational language are implemented in future mental health chatbots, ensuring agency and ethical accountability. Third, although participants engaged with the chatbots for 4 weeks, the intensity and depth of bonding varied across individuals. We did not conduct a formal quantitative analysis linking relationship types or specific themes to changes in participants’ well-being, such as WHO-5 scores. Future studies could combine qualitative analysis with outcome measures to examine whether certain relational patterns are associated with better emotional well-being or greater benefit over time. Fourth, our sample primarily consisted of university students and young adults in New Zealand, many of whom had backgrounds in psychology or computer science. This may have influenced their perspectives due to prior exposure to mental health concepts or AI technologies. Most of whom were digitally literate and relatively well-educated, which limits the generalizability of findings to broader or less digitally fluent populations. Cultural expectations, clinical needs, and access to technology may differ across age groups, education levels, and geographic regions. Future research should include more diverse participants, such as individuals from clinical populations, older adults, or communities outside of urban Western and Asian settings. Finally, participants also mentioned elements such as tone of voice, color schemes, emojis, and overall visual design. However, these features were not consistently linked to how users perceived their relationship with the chatbots. Most participants did not view these aspects as playing a significant role in building or deepening the bond. Therefore, this study focused on relational dimensions—such as empathy, responsiveness, and autonomy—which appeared more directly tied to a sense of bonding. Future research could further explore how design aesthetics and interaction style might support or hinder relationship development in different contexts.

### Conclusions

The potential effectiveness of the DTA is promising. But what aspects influence its success, and how do people perceive it? Our research explored the human relationship with mental health chatbots and identified six key themes that shape relationship-building. Diverse preferences for conversational control impact the bond: some preferred to lead, others to be led, and there is a desire for a more dynamic balance of both. While many favored free-text input, our findings highlighted the importance of matching input methods to preferences and adapting them to different situations. In this study, people sought not just basic empathetic care, but deeper, more consistent emotional support. Relationships began to form through routine check-ins and became stronger with supportive activities, especially those that involve collaboration. Recalling past conversations made interactions feel more personal and human-like, further enhancing the bond. Our findings expand the DTA framework by providing new insights into how relationships with mental health chatbots develop. These findings have implications for the design of chatbots that foster stronger connections.

## Appendix

Multimedia Appendix 1 Interview guide.

Multimedia Appendix 2 COREQ checklist.

## Abbreviations

AI: artificial intelligence
ARM: Agnew Relationship Measure
COREQ: Consolidated Criteria for Reporting Qualitative Research
DTA: digital therapeutic alliance
D-WAI: Digital Working Alliance Inventory
EU: European Union
HCI: human-computer interaction
mARM: mobile Agnew Relationship Measure
TA: therapeutic alliance
WAI: Working Alliance Inventory
WAI-S: Working Alliance Inventory-Short
WHO-5: World Health Organization—Five Well-Being Index
