# Supplementary material for: The Digital Therapeutic Alliance With Mental Health Chatbots: Diary Study and Thematic Analysis
Source: JMIR Ment Health. 2025 Oct 10;12:e76642. doi: 10.2196/76642 (PMC12552820; doi:10.2196/76642)
Supplement: Multimedia Appendix 1 [file mental_v12i1e76642_app1.pdf]

Interview Questions

Week 1

Daily Use and Experience:

1. Can you walk me through a typical day when you use the app?
2. When do you usually use the app? Is it at specific times or moments?
3. How do you decide when it's time to use the app?
4. What has your overall experience been like using this app?
5. Can you share 1-2 memorable experiences with the app? What stood out to you about those moments? How did you feel during that time?
6. Can you describe your most recent interaction with the app? How did it make you feel, and what parts of that experience stood out to you?
7. Tell me more about your feelings while using the app.

Review of Screenshots:

8. Let's go through these screenshots. Can you explain what's happening in this one?
9. How did you feel when this happened? What did you do next? How did you feel during that time?
10. When something stood out to you or caught your attention in the app, what was it, and why? How did you feel during that time?

If participants shared the any examples of below aspects, then ask, if not, leave those questions to week2

Bond/Connection:

11. If you felt a bond with the app, can you describe what that bond was like?
12. Can you recall specific moments or times when you felt connected to the app?
13. How did this bond develop over time?
14. What qualities do you think led to different levels of bonding?

Expressiveness/Communication:

15. Can you share an example of a time when you felt expressive while using the app? What made that moment special?
16. Have you ever found it challenging to express yourself while using the app? Can you describe those moments?

Engagement:

17. Can you describe a time when you felt particularly engaged with the app?
18. Were there any moments when you struggled to stay engaged with the chatbot? What made it difficult?

Trust:

19. If you experienced a sense of trust with the app, how would you describe it?
20. Can you recall moments when trust felt strong or when it was difficult to trust the app?

Week 2

Overall Relationship:

1. How would you describe the relationship between you and this chatbot? Why?
2. How have you been feeling about the app over the past week?
3. Can you recall anything unexpected or surprising that happened in the last two weeks? Why did it stand out to you?
4. Comparing the first and second week, how have your feelings about the app changed? Have they become more positive or negative?

Daily Use and Experience:

5. Can you walk me through a typical day when you use the app?
6. When do you usually use the app? Is it during specific times or in certain moments?
7. How do you decide when it’s time to use the app?
8. What has your overall experience been like using this app?
9. Can you share 1-2 memorable experiences you’ve had with the app? What made those moments stand out, and how did you feel during them?
10. What was your most recent interaction with the app like? How did it make you feel, and what stood out to you?
11. Can you tell me more about your feelings while using the app?

Review of Screenshots:

12. Let’s go through these screenshots together. Can you explain what’s happening in this one?
13. How did you feel when that happened? What did you do next?
14. When something caught your attention in the app, what was it, and why did it stand out? How did you feel at that time?

Bond/Connection:

15. Do you feel a bond with this app?
- If not, could you explain why?
- If yes, can you describe what that bond feels like?
16. Can you recall any specific moments when you felt connected to the app?
17. How has this bond developed over time?
18. What qualities do you think created different levels of bonding with the app?

Expressiveness/Communication:

19. Have you ever felt “expressive” when using this app?
- If not, why do you think that is?
- If yes, can you share a time when you felt expressive while using the app? What made that moment stand out?
20. Can you recall any moments when you were motivated to express more with the chatbot or moments when you felt unmotivated to express yourself? Can you give an example of each? How did those situations make you feel?
21. Have you ever found it challenging to express yourself while using the app? Can you describe those moments?

Engagement:

22. Have you ever felt “engaged” when using the app?
- If not, why do you think that is?
- If yes, can you describe a time when you felt particularly engaged with the app?
23. Were there moments when you struggled to stay engaged with the chatbot? What made it difficult to stay engaged?

Trust:

24. Have you ever felt “trust” when using this app?
- If not, why do you think that is?
- If yes, how would you describe the sense of trust you’ve felt?
25. Can you recall any moments when trust felt particularly strong or when it was difficult to trust the app?

Week 3

Daily Use and Experience:

1. Can you walk me through a typical day when you use the app?
2. When do you usually use the app? Is it at specific times or moments?
3. How do you decide when it's time to use the app?
4. What has your overall experience been like using this app?
5. Can you share 1-2 memorable experiences with the app? What stood out to you about those moments? How did you feel during that time?
6. Can you describe your most recent interaction with the app? How did it make you feel, and what parts of that experience stood out to you?
7. Tell me more about your feelings while using the app.

Comparing Chatbot Experiences:

8. How does your experience with this chatbot compare to any previous chatbots you've used?
9. Did you notice any differences in how you interacted with Woebot and Wysa?
10. How did the interactions between Woebot and Wysa differ for you?
11. What interactions or experiences with this chatbot stand out as different from the others?
12. Which interactions or experiences do you prefer, and why?
13. How did each of these interactions or experiences make you feel, and what influenced your preference?

Review of Screenshots:

14. Let's go through these screenshots. Can you explain what's happening in this one?
15. How did you feel when this happened? What did you do next? How did you feel during that time?
16. When something stood out to you or caught your attention in the app, what was it, and why? How did you feel during that time?

If participants shared the any examples of below aspects, then ask, if not, leave those questions to week4

Bond/Connection:

17. If you felt a bond with the app, can you describe what that bond was like?
18. Can you recall specific moments or times when you felt connected to the app?
19. How did this bond develop over time?
20. What qualities do you think led to different levels of bonding?

Expressiveness/Communication:

21. Can you share an example of a time when you felt expressive while using the app? What made that moment special?
22. Have you ever found it challenging to express yourself while using the app? Can you describe those moments?

Engagement:

23. Can you describe a time when you felt particularly engaged with the app?
24. 19. Were there any moments when you struggled to stay engaged with the chatbot? What made it difficult?

Trust:

25. If you experienced a sense of trust with the app, how would you describe it?
26. Can you recall moments when trust felt strong or when it was difficult to trust the app?

Week 4

Overall Relationship:

1. How would you describe the relationship between you and this chatbot? Why?
2. How have you been feeling about the app over the past week?
3. Can you recall anything unexpected or surprising that happened in the last two weeks? Why did it stand out to you?
4. Comparing the first and second week, how have your feelings about the app changed? Have they become more positive or negative?

Daily Use and Experience:

5. Can you walk me through a typical day when you use the app?

6. When do you usually use the app? Is it during specific times or in certain moments?
7. How do you decide when it's time to use the app?
8. What has your overall experience been like using this app?
9. Can you share 1-2 memorable experiences you've had with the app? What made those moments stand out, and how did you feel during them?
10. What was your most recent interaction with the app like? How did it make you feel, and what stood out to you?
11. Can you tell me more about your feelings while using the app?

Review of Screenshots:

12. Let's go through these screenshots together. Can you explain what's happening in this one?
13. How did you feel when that happened? What did you do next?
14. When something caught your attention in the app, what was it, and why did it stand out? How did you feel at that time?

Bond/Connection:

15. Do you feel a bond with this app?

- If not, could you explain why?
- If yes, can you describe what that bond feels like?

16. Can you recall any specific moments when you felt connected to the app?
17. How has this bond developed over time?
18. What qualities do you think created different levels of bonding with the app?

Expressiveness/Communication:

19. Have you ever felt "expressive" when using this app?

- If not, why do you think that is?
- If yes, can you share a time when you felt expressive while using the app? What made that moment stand out?

20. Can you recall any moments when you were motivated to express more with the chatbot or moments when you felt unmotivated to express yourself? Can you give an example of each? How did those situations make you feel?
21. Have you ever found it challenging to express yourself while using the app? Can you describe those moments?

Engagement:

22. Have you ever felt "engaged" when using the app?

- If not, why do you think that is?
- If yes, can you describe a time when you felt particularly engaged with the app?

23. Were there moments when you struggled to stay engaged with the chatbot? What made it difficult to stay engaged?

Trust:

24. Have you ever felt “trust” when using this app?

- If not, why do you think that is?
- If yes, how would you describe the sense of trust you’ve felt?

25. Can you recall any moments when trust felt particularly strong or when it was difficult to trust the app?

Comparing Chatbot Experiences:

26. How does your experience with this chatbot compare to any previous chatbots you’ve used?
27. Did you notice any differences in how you interacted with Woebot and Wysa?
28. How did the interactions between Woebot and Wysa differ for you?
29. What interactions or experiences with this chatbot stand out as different from the others?
30. Which interactions or experiences do you prefer, and why?
31. How did each of these interactions or experiences make you feel, and what influenced your preference?
